# Supplementary material for: Splice-Junction-Based Mapping of Alternative Isoforms in the Human Proteome
Source: Cell Rep. Author manuscript; Available in PMC 2020 Jan 15. (PMC6961840; doi:10.1016/j.celrep.2019.11.026)
Supplement: 2 [file NIHMS1546469-supplement-2.zip › DF1/PXD006675/RightVentricle/RightVentricle_56_ADCK1_VSWTSMALAASGIYFYSNK.pdf]

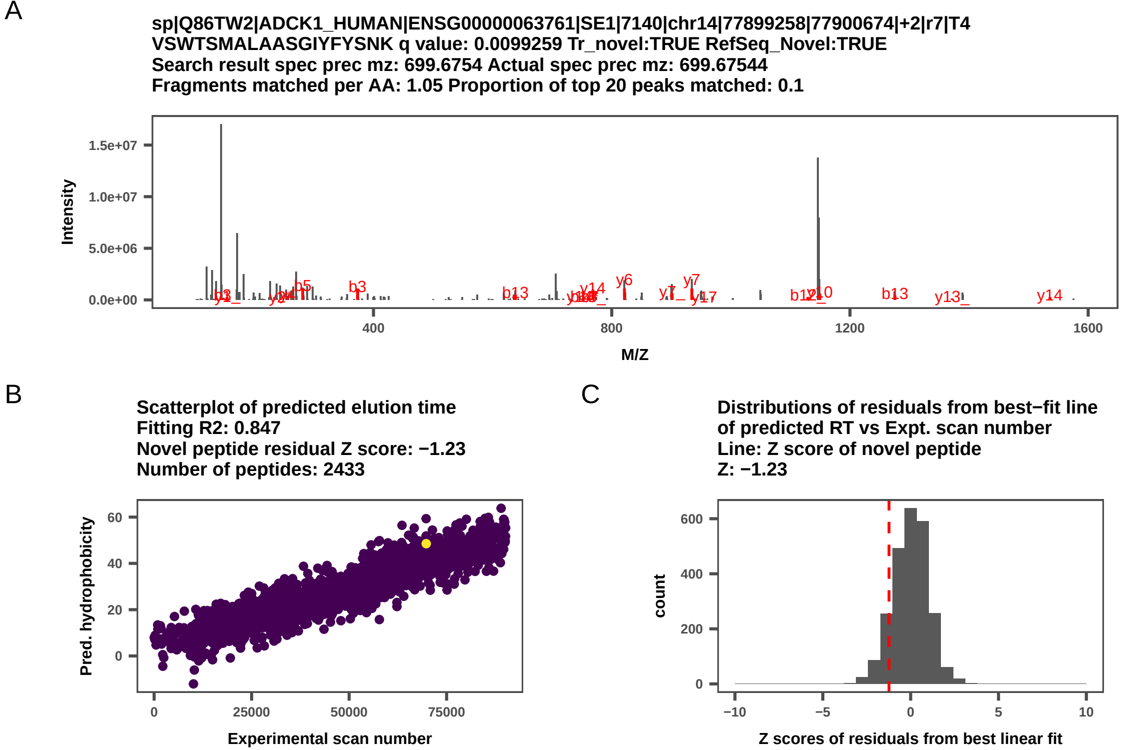
A
sp|Q86TW2|ADCK1_HUMAN|ENSG00000063761|SE1|7140|chr14|77899258|77900674|+2|r7|T4
VSWTSMALAASGIYFYSNK q value: 0.0099259 Tr_novel:TRUE RefSeq_Novel:TRUE
Search result spec prec mz: 699.6754 Actual spec prec mz: 699.67544
Fragments matched per AA: 1.05 Proportion of top 20 peaks matched: 0.1
Intensity
1.5e+07
1.0e+07
5.0e+06
0.0e+00
b5
b3
b13
y6
y7
y17
y13_
y14
400
800
1200
1600
M/Z
B
Scatterplot of predicted elution time
Fitting R2: 0.847
Novel peptide residual Z score: −1.23
Number of peptides: 2433
Pred. hydrophobicity
60
40
20
0
0
25000
50000
75000
Experimental scan number
C
Distributions of residuals from best−fit line
of predicted RT vs Expt. scan number
Line: Z score of novel peptide
Z: −1.23
count
600
400
200
0
−10
−5
0
5
10
Z scores of residuals from best linear fit
